# The Role of FTO Risk Haplotype in Overweight/Obesity and Lipid Parameters-Results From the Central China Population Study

**DOI:** 10.1155/2024/8062791

**Published:** 2024-10-24

**Authors:** Meiwei Ning, Lin Chen, Yuxue Wang, Aohong Xu, Rong Zeng, Huan Zhang, Boda Wang, Xiang Liu

**Affiliations:** ^1^Department of Laboratory Medicine, Hubei University of Chinese Medicine, Wuhan, Hubei, China; ^2^Heilongjiang University of Chinese Medicine Jiamusi College, Jiamusi, China; ^3^Clinical Laboratory, Huangjiahu Hospital of Hubei University of Chinese Medicine, Wuhan, China

**Keywords:** FTO, glucose and lipid parameters, haplotype, overweight/obesity, polymorphisms

## Abstract

**Background:** Fat mass and obesity-associated gene (FTO) genes rs9939609 is strongly associated with obesity and rs17817449 is an important and potential gene for obesity, have been well established. We aim to evaluate the relationship between FTO gene and overweight/obesity and confirm the influence of obesity on glucose and lipid metabolism parameters.

**Methods:** We investigated 183 normal weight subjects and 193 individuals with overweight/obesity. Firstly, the effect of overweight/obesity on glucose and lipid metabolism parameters was analyzed. Then, the FTO genes rs9939609 and rs17817449 were counted to explore whether polymorphisms were associated with overweight/obesity and metabolic parameters.

**Results:** Significant differences existed in glucose and lipid parameters between the group with overweight/obesity and control group. The rs9939609 and rs17817449 were strongly correlated with overweight/obesity. Haplotype analysis revealed that GA and GT haplotypes had 2.99 and 1.81 fold risk of overweight/obesity. FTO polymorphism also has effects on glucose and lipid metabolism parameters.

**Conclusions:** There is a linkage imbalance between rs9939609 and rs17817449 in a Central China general population cohort, which also reflected the influence of FTO gene on the risk of overweight/obesity and total cholesterol (TC), triglyceride (TG), and high-density lipoprotein (HDL) disorders. The new findings could provide new clues to predict obesity and metabolic diseases.

## 1. Introduction

Obesity is now recognized to be important in the pathogenesis of chronic serious diseases, including metabolic diseases such as cardiovascular disease and type 2 diabetes [1], respiratory diseases such as coronavirus disease-2019 [2], and even various cancers [3]. Genome-wide association analysis (GWAS) revealed the first genetic factor closely related to human obesity: Fat mass and obesity-associated (FTO) gene [4]. It regulates gene expression through a transformation of the methylation and demethylation states of nucleic acids [5, 6], which is highly expressed in hypothalamus tissue [7]. These biological characteristics suggest that FTO may play a role in managing energy balance via controlling food intake, energy expenditure, and fat metabolism. And excess energy accumulation leads to obesity [8–10].

FTO polymorphisms rs9939609 was the first identified locus that has been confirmed to be strongly associated with obesity in numerous large-scale trials [11–14]. In subsequent experiments with other polymorphism in the first intron of FTO, a link between the variations of rs17817449 and higher BMI, waist circumference, and fat mass was observed in the Europe cohort [12, 15, 16]. But this association did not exist in studies of repetitive rs17817449 variants associated with obesity-related phenotypes in Oceania and African Americans [17, 18]. Recent studies have broadened our understanding of the FTO gene, showing that rs9939609 and rs17817449 have strong linkage imbalances and that haplotype enhances susceptibility to obesity [19]. Previous studies have clarified that obesity is significantly related to high glucose, high total cholesterol (TC), high triglyceride (TG), high low-density lipoprotein (LDL), and low high-density lipoprotein (HDL) [20–22]. Since the FTO gene is a genetic factor of obesity, researchers have focused on a potential association between dyslipidemia and the FTO gene [23, 24]. Lukasova et al. [25] further proposed the important role of rs9939609 (A) and rs17817449 (G) haplotypes in elevating glucose, TC, and LDL. However, very little is known about how rs17817449 and combine with the common variant of rs9939609 might contribute differentially to obesity and metabolic parameters.

Therefore, we plan to evaluate whether rs9939609 and rs17817449 genes may predict overweight/obesity to verify the influence of obesity on glucose and lipid parameters. Subsequently, we explore the hypothesis that FTO may have some influence on glucose and lipid parameters.

## 2. Methods

### 2.1. Subject

The data collected in this study were collected from the population who came to Huangjiahu Hospital for a physical examination in May 2021. The study included 376 participants (193 individuals were overweight/obesity and 183 individuals were normal body weight). Because of the large age span of participants, we regard 40 years old as a dividing line. 181 participants (74 individuals were overweight/obesity and 107 individuals were normal body weight) with less than 40 years old and 195 participants (119 individuals were overweight/obesity and 76 individuals were normal body weight) with over 40 years old. To ensure the accuracy of this study, we selected healthy subjects with no genetic relationship to each other; we excluded participants (1) who had any acute or chronic medical conditions, (2) had no significant fluctuation in weight in the near term, (3) pregnant and breastfeeding woman, and (4) who were taking any medications that might affect metabolic function. For this study, we obtained ethical committee approval, and informed consent was also obtained from all subjects before participation in the study. The approval number is [2018] IEC (010).


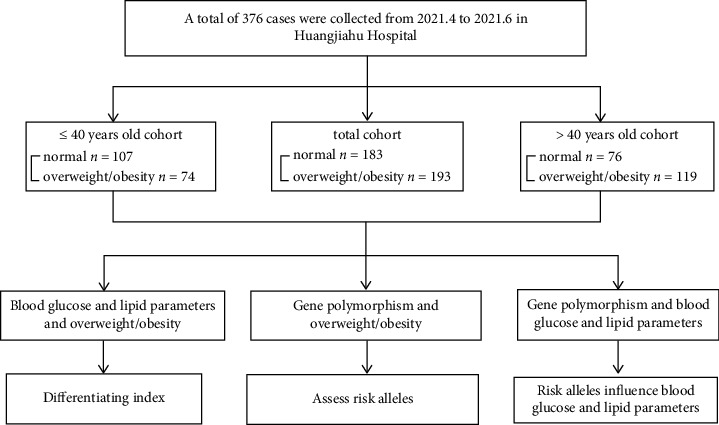


### 2.2. Body Mass Index (BMI) Calculation and Grouping

BMI is defined as weight in kilograms (kg) divided by height in meters squared (m^2^), which was a commonly used international standard to measure the whether people healthy and degree of body fat and thinness [26]. To obtain an accurate BMI value, the height and weight of participants were measured by specially trained professionals of the university hospital following standard procedures [26]. Body weight was measured by wearing light and removing shoes. The study groups were classified into two groups according to their BMI: normal weight (BMI 18.5–24 kg/m^2^), and overweight/obesity (BMI > 24 kg/m^2^) [4].

### 2.3. Measurement of Glucose and Lipid Parameters

Blood samples were collected after fasting for 10–12 h, and the levels of glucose, TC (mmol/L), TG (mmol/L), LDL (mmol/L), and HDL (mmol/L) in serum were measured using an automatic analyzer (COBAS C501; Roche Diagnostics, United States). Glucose (mmol/L) was measured by the HK method, TC was measured by the cholesterol-oxidase method, TG was measured by the GPO-POD method, and LDL and HDL were measured by the homogeneity method.

### 2.4. Genotyping

Genomic DNA was extracted from blood using the blood DNA extraction kit (Tiangen Biochemical Technology Co., Ltd., Beijing), and genotyping of both polymorphisms in the FTO gene was carried out using the Sanger sequencing method. Detection of rs9939609 and rs17817449 was performed using previously published primer sets [27], it was synthesized by Wuhan Shenggong Bioengineering Company. rs9939609 forward primer: 5′-TCC CAC TCC ATT TCT GAC TGT TAC -3′ and Reverse primer: 5′-AAT TCA AAA CTG GCT CTT GAA TGA -3′, While the sequence of rs17817449 forward 5′-GTG CCT TAC GGT GAA GAG GA-3′, and reveres primer was 5′-TGT ACC CGA AAT GAG TCT TCG-3′.

The amplification of the two SNPs was performed in a total volume of 20 *μ*L which contained 14.3 *μ*L of ddH_2_O, 2 *μ*L of Buffer, 0.8 *μ*L of MgCl_2_, 0.4 *μ*L of dNTP, 0.4 *μ*L of each primer, 0.1 *μ*L of Taq DNA polymerase, and 1 *μ*L of DNA template (All reagents were from Beijing TIANGEN BIOTECH CO., LTD).

The cycling conditions for rs9939609 and rs17817449 were 94°C for 3 min, followed by 30 cycles of 94°C for the 30s, 55°C for 30s, 72°C for 30s, and a final extension of 72°C for 10 min, respectively.

### 2.5. Statistical Analysis

SPSS 23.0 was used for statistical analysis. The measurement data of normal distribution were expressed as mean ± standard deviation. The Student's *T*-test was used to compare parameters between two groups. The measurement data of non-normal distribution were expressed as M (P25, P75), and the difference between the two groups was compared by Mann–Whitney *U* test. Allele frequencies in case and control of FTO rs9939609 and rs17817449 concerning BMI status were assessed for the association by Pearson's chi-square test. Risk assessment was performed using Binary logistic regression analysis and linear regression analysis coefficients. Haplotypes were obtained and their associations with obesity were verified by the *χ*^2^ test (OR was calculated). The haplotype analysis between the two SNPs was determined using the software (haplotype analysis). *p* < 0.05 was considered a statistical significance.

## 3. Results

### 3.1. The Difference in Characteristics Between the Control Group and the Overweight/Obesity Group

The clinical characteristics and glucose and lipid parameters of 376 subjects are shown in Table 1. The group with overweight/obesity had significantly higher mean values of glucose (*p* < 0.05), TG (*p* < 0.05), and LDL (*p* < 0.05) than the control group, whereas the mean values of HDL (*p* < 0.05), were significantly higher in the control group compared to group with overweight/obesity. After the subjects were stratified by sex and age, the differences between different groups are shown in Table 2.

### 3.2. FTO Frequencies and Hardy–Weinberg Equilibrium

The genotypic and allelic frequencies are summarized in Tables 3 and 4. Genotypes of loci rs9939609 and rs17817449 polymorphisms were in Hardy–Weinberg equilibrium (*p* > 0.05) among both overweight/obesity and control groups. The frequencies of these 3 genotypes and allele frequencies of loci rs9939609 and rs7817449 were comparable between the overweight/obesity and control groups.

### 3.3. Influence of FTO Variants on Obesity Risk

Binary logistic regression was subsequently applied for risk assessment of overweight/obesity (Table 5). We could conclude from the tables that TA genotype of loci rs9939609 had a remarkable effect on overweight/obesity regardless of whether other factors are corrected. Meanwhile, TA + AA genotype has a significant effect on overweight/obesity in the dominant model. Subjects carrying the rs9939609 (A) allele were 2.48 times more likely to be overweight/obesity. Individuals carrying the rs17817449G risk allele had an extraordinary impact on overweight/obesity in all model. Subjects carrying at least one rs17817449 (G) had a 2.15 times higher risk for developing this phenotype.

### 3.4. Differences in Glucose and Lipid Parameters Between FTO Polymorphisms

The comparison of glucose and lipid parameters between all genotypes of FTO polymorphisms is shown in Table 6. Significant differences were diverse in HDL and TG levels in all cohorts of rs9939609 (*p* < 0.05), Glu was statistically different in total cohorts and (*p* < 0.001) and ≤ 40 years cohorts (*p* < 0.001), TC and LDL were significant differences in > 40 years cohorts (*p* < 0.05). About rs17817449, significant differences existed in HDL and TG among all cohorts (all *p* < 0.001).

### 3.5. Regression Coefficients of FTO Polymorphism and Lipid Parameters

**Figure 1:**
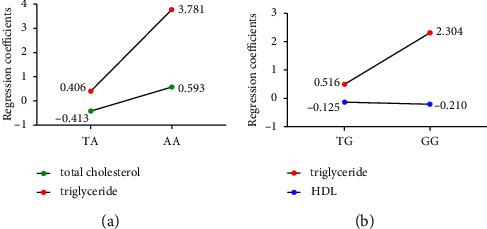
Regression coefficients of FTO risk allele genotypes and lipid parameters. (a) regression coefficient of rs9939609 on TC and TG and (b) regression coefficient of rs17817449 on TG and HDL. P adjusted for sex, age, weight, and BMI.

Linear regression analysis was then used for evaluating the correlation coefficient between FTO gene polymorphism and lipid parameters; TC, TG and HDL were used as dependent variables, and genotype carrying risk allele was used as the independent variable (Figure 1). The TA and AA genotype of loci rs9939609 was positively correlated with TG, but the effect of TA and AA on TC was different. The TG and GG genotypes of loci 17,817,449 was positively correlated with TG, but negatively correlated with TC.

### 3.6. Haplotype and Linkage Disequilibrium

Haplotype group analyses were performed using FTO rs9939609 and rs17817449, our analysis demonstrated that FTO rs9939609 and rs17817449 are in linkage disequilibrium (D′ = 1.0; *r*^2^ = 0.329; LOD = 34.76). Haplotype analysis revealed three haplotypes in the study, with GA (12.1%), GT (17.4%), and TT (70.5%) being the most common. As shown in Table 7, compared TT haplotypes were significantly associated with overweight/obesity and the risk of increased TC, TG, and decreased HDL.

## 4. Discussion

This study evaluated the effects of the FTO gene on overweight/obesity and glucose and lipid parameters in the Central China population. Through our study of data collected from participants, this study proved that the haplotype of rs9939609 and rs17817449 were closely related to overweight/obesity. Overweight/obesity had a significant influence on glucose, TG, HDL, and LDL. Interestingly, rs9939609 and rs17817449 even haplotype contributed to significant changes in TC, TG, and HDL.

In the current study, we found that rs9939609 and rs17817449 were strongly correlated with individuals of overweight/obesity. The rs9939609 was the most widely replicated locus, its association with obesity risk has been tested by many researchers in different populations and GWAS has also repeatedly confirmed the risk role of rs9939609 in obese phenotypes [14, 28]. Subjects carrying the rs9939609 (A) allele were 2.48 times more likely to be overweight/obesity. Again, the risk of rs9939609 polymorphism for obesity was determined. The allele frequency of rs9939609 (A) was in the overweight/obesity group and the control group was 16.8% and 7% consisting of 13.2% of the recently reported Han Chinese population [29]. It was also significantly lower than the 41% risk allele frequency previously found in European populations [30]. Compared with the TT genotype, the TA genotype had a significantly higher risk of overweight/obesity, while the AA genotype had no statistically significant effect on overweight/obesity. This may be because the number of AA genotype carriers is too small, making statistical data deviation.

rs17817449 was solely reported separately in FTO polymorphism studies, in which the risk allele G may regulate telomere shortening via nucleic acid demethylation [31]. Telomere shortening also reported participating in obesity and related diseases [32]. From the reports focusing on the relationship between rs17817449 and obesity, studies have validated the association of rs17817449 (G) with obesity phenotypes studied on European Population [33], Mexican women [34], Korean [35], Thai [36], and Egyptian population with obesity [37]. However, studies on Oceanic [18], the children of Egypt [38], and African Americans populations [17] were irrelevant. As the risk allele of rs17817449, the existence of gene heterogeneity made it also express T [19, 39, 40]. Our study also proved that rs17817449 was a risk gene for overweight and obesity in the Chinese population. The risk allele (G) frequency was 29.5%, well below the 62% risk allele frequency in the European adult [12], whereas much higher than 14.7% reported in Korea [35]. These results suggested ethnic and geographic differences in the frequency of this FTO locus. In addition, our results showed a strong linkage disequilibrium in both FTO variants. Compared to the common TT haplotype, GT increased the risk of overweight/obesity development by 1.81 times, and GA increased the risk by up to 2.99 times. It is accorded with the haplotype mentioned in Brazilian with obesity [19].

Meanwhile, our results showed that obesity was strongly correlated with high glucose and dyslipidemia. One study with a sample size of 4809 Turkish adults over the age of 20 and the other including 11,018 Chinese adults with an average age of 46, both found that the prevalence of high TC, TG, LDL, and low HDL increased steadily with the increase of body composition [41, 42]. Analysis of Korean adolescents found that BMI was positively correlated with glucose and TG [43]. Moreover, a study pointed out that TG was the strongest predictor of obesity among Brazilian university students [44]. Our results were consistent with previous studies. These results confirmed the important role of overweight/obesity in abnormal glucose and lipid metabolism. At the same time, adipose tissue is involved in a variety of metabolic activities, and a variety of adipokines secreted by adipocytes also interact with metabolic parameters [45]. In our study, the increase of glucose level was accompanied by the decrease of HDL level, and the decrease of HDL concentration was accompanied by TG concentration. This is because the high concentration of glucose in plasma transfers cholesterol from HDL to very LDL particles [46]. The further decrease of HDL concentration is due to the conversion of hepatic lipase into smaller particles, which can be quickly removed from the plasma [47]. The resulting VLDL particles form cholesterol ester depletion small and dense LDL particles, which are absorbed by arterial wall macrophages, leading to atherosclerosis and other diseases [48].

Given the relationship between obesity and FTO and metabolic parameters, we also hypothesized that FTO affects metabolic parameters. As we expected, the three cohorts of rs9939609 showed significant differences in glucose and lipid parameters among genotypic subgroups. After correcting gender, age, weight, and BMI, this relationship still exists. Interestingly, the AA and TA genotypes have different effects on TG levels. Similarly, in a European multicohort study, Rachel et al. [49] found that the rs9939609 A allele acted on high glucose, TG, and low HDL cholesterol, independent of BMI. Other subsequent studies that have been replicated in patients with diabetes suggested that fluctuations in TC, TG, and HDL levels were associated with the occurrence of the rs9939609 A allele [50, 51]. Other studies of polymorphism in rs9939609 showed variation was a risk factor for HDL decline among acromegaly patients and males with cardiovascular disease [52, 53]. Duicu et al. [33] also supplemented that rs9939609 (AA) significantly increased LDL levels in the healthy crowd. Contrary to our results, some studies found no association between rs9939609 polymorphisms and glucose and lipid parameters [39, 54]. About rs17817449, after adjusting for confounding factors, the risk genes were only correlated with high TG and low HDL. Moreover, the haplotype of GT has a strong effect on TC, TG, and HDL, while GA has an extraordinary effect on TG and HDL. All these findings indicated the essential role of FTO polymorphism in TC, TG, and HDL level disorders and the process is independent of BMI. The potential relationship between rs17817449 SNP and glucose, TC, and TG were also explored [33]. Fonseca et al. [19] repeated that the rs17817449 risk allele affects TG levels in extremely Brazilians with obesity. The authors indicated that rs9939609 and rs17817449 were involved in regulating fat. Yet at the same time, other fact-seeking studies have failed to find correlations [35, 38]. The reasons for the differences in these results can be explained by different methodological designs and experimental populations. In addition, geographical differences cannot be ignored.

Although the exact mechanism of the association between FTO gene and glucose and lipid parameters has not been agreed upon, several recent studies have helped to broaden our understanding. The FTO gene encodes 2-oxoglutarate (2-OG) Fe (II)-dependent dioxygenase, whose mRNA and expressed proteins perform a variety of functions to maintain body function [55, 56]. Some studies have concluded that variations in the first intron of FTO cause differences in methylation capacity, and that methylation status regulates the development of obesity and related diseases [57, 58]. Mizuno et al. record that FTO may regulate lipid metabolism by altering m6A modification status and lipid metabolism-related gene expression in vivo. And it is possible to regulate the expression of gluconeogenic genes by changing the activity of transcription factors and their interactions [59]. As emerging research suggests, higher expression and methylation of the FTO gene, lowers the levels of apelin, resistin, and leptin receptor in children. This suggests that FTO overexpression may be responsible for adipose tissue enlargement and energy excess by directly regulating adipose hormones such as apelin, resistance, and leptin receptors [60]. Likewise, the Sirtuin 1 gene, which slows aging and controls appetite, also played an important role in the development of obesity and the disturbance of glucose and lipid metabolism. Sirtuin1 has been shown to slow the aging process in rodents by limiting caloric consumption [61–63]. So in terms of energy metabolism, what kind of crossover and coordination exists between FTO gene and Sirtuin 1 gene regulation is unknown. Then, rs17817449 (G), rs9939609 (A), and rs8050136 (A) have fairly strong LD pairs with each other [64]. The rs17817449 and rs8050136 were located in the recognition sequence of a cultlike-1 transcription factor in human fibroblast DNA [65]. Recently, rs8050136 is strongly correlated with retinoblastoma-like 2 expression [66]. These may help explain the effects of FTO polymorphism on glucose and lipid parameters observed. However, further mechanistic studies are needed to determine whether FTO polymorphism functionally affects these processes in human metabolism.

Our study highlights the role of haplotypes rs9939609 and rs17817449 and the risk allele in overweight and obesity in the Central China population. Our results prove the important value of both polymorphisms and the combination of risk alleles in the increase of TC and TG and the decrease of HDL.

## Figures and Tables

**Table 1:** Characteristic of control and overweight/obesity population.

Parameters	Control	Overweigh/obesity	*p*
Gender (F/M)	100/83	88/105	0.080
Weight (kg)	55 (51∼61)	74 (67∼80)	<0.001
BMI (kg/m^2^)	21.19 (19.51∼22.53)	25.71 (24.76∼27.22)	<0.001
Glu (mmol/L)	5.11 (4.88∼5.40)	5.27 (4.99∼5.71)	<0.001
TC (mmol/L)	4.74 (4.22∼5.33)	4.96 (4.42∼5.51)	0.095
TG (mmol/L)	0.86 (0.65∼1.24)	1.40 (0.97∼2.02)	<0.001
HDL (mmol/L)	1.53 ± 0.28	1.32 ± 0.26	<0.001
LDL (mmol/L)	2.73 (2.26∼3.22)	3.13 (2.59∼3.58)	<0.001

Abbreviations: BMI, body mass index; Glu, glucose; HDL, high-density lipoprotein cholesterol; LDL, low-density lipoprotein cholesterol; TC, total cholesterol; TG, triglyceride.

**Table 2:** Difference in characteristic of study population.

		≤ 40	> 40	*p*
Weight (kg)	Man	74.78 ± 11.32	74.29 ± 9.97	0.771
	Woman	55 (50∼59.5)	58 (53∼62.5)	0.009
	*p*			<0.001
BMI (kg/m^2^)	Man	24.67 ± 3.02	25.18 ± 2.83	0.267
	Woman	21.23 ± 2.99	22.72 ± 2.98	<0.001
	*p*			<0.001
Glu (mmol/L)	Man	5.12 ± 0.41	5.48 ± 0.72	<0.001
	Woman	5.10 (4.91∼5.35)	5.24 (4.96∼5.54)	0.068
	*p*			0.285
HDL (mmol/L)	Man	1.28 ± 0.22	1.26 ± 0.24	0.667
	Woman	1.53 ± 0.27	1.54 ± 0.27	0.951
	*p*			<0.001
LDL (mmol/L)	Man	2.99 ± 0.68	3.14 ± 0.66	0.158
	Woman	2.56 (2.19∼3.08)	3.02 (2.45∼3.48)	<0.001
	*p*			0.001
TC (mmol/L)	Man	4.82 ± 0.86	4.96 ± 0.85	0.282
	Woman	4.62 ± 0.77	5.17 ± 0.96	<0.001
	*p*			0.906
TG (mmol/L)	Man	1.37 (0.92, 2.08)	1.4 (1.04, 1.96)	0.825
	Woman	0.72 (0.56∼0.985)	1.10 (0.76∼1.51)	<0.001
	*p*			<0.001

Abbreviations: BMI, body mass index; Glu, glucose; HDL; high-density lipoprotein cholesterol; LDL, low-density lipoprotein cholesterol; TC, total cholesterol; TG, triglyceride.

**Table 3:** Comparison of rs9939609 genotype and allele frequency of FTO gene.

	Locus	Genotype			*χ* ^2^	*p*	Allele		*χ* ^2^	*p*
		TT (%)	TA (%)	AA (%)			*T* (%)	*A* (%)		
Overweight/Obesity	FTO rs9939609	135 (70.0)	51 (26.4)	7 (3.6)	16.00	0.001	321 (83.2)	65 (16.8)	16.74	0.001
Control		159 (86.9)	22 (12)	2 (1.1)			340 (93)	26 (7)		

**Table 4:** Comparison of genotype and allele frequencies of FTO gene rs17817449.

	Locus	Genotype			*χ* ^2^	*p*	Allele		*χ* ^2^	*p*
		TT (%)	TG (%)	GG (%)			*T* (%)	*G* (%)		
Overweight/Obesity	FTO rs17817449	72 (37.30)	97 (50.30)	24 (12.40)	25.57	<0.001	241 (62.40)	145 (37.60)	24.66	<0.001
Control		112 (61.20)	65 (35.50)	6 (3.30)			289 (79)	77 (21)		

**Table 5:** Effect of FTO rs9939609 and rs178174 polymorphism on overweight/obesity.

	Total cohort		≤ 40 cohort		> 40 cohort	
	Model 1 OR (95CI)	Model 2 OR (95CI)	Model 1 OR (95CI)	Model 2 OR (95CI)	Model 1 OR (95CI)	Model 2 OR (95CI)
rs9939609						
TT	Ref					
TA	2.73⁣^∗^(1.57–4.73)	2.46⁣^∗^(1.40–4.31)	2.77⁣^∗^(1.21–6.35)	2.65⁣^∗^(1.14–6.12)	2.41⁣^∗^(1.14–5.13)	2.31⁣^∗^(1.08–4.95)
AA	4.12 (0.84–20.18)	4.21 (0.85–20.94)	7.17 (0.78–65.81)	6.68 (0.72–61.69)	2.34 (0.24–23.05)	2.31 (0.22–22.86)
Dominant model						
TT	Ref					
TA + AA	2.85⁣^∗^(1.68–4.83)	2.61⁣^∗^(1.53–4.47)	3.14⁣^∗^(1.43–6.88)	2.99⁣^∗^(1.35–6.60)	2.41⁣^∗^(1.11–4.99)	2.31⁣^∗^(1.11–4.82)
Recessive model						
TT + TA	Ref					
AA	3.41 (0.69–16.61)	3.56 (0.71–17.71)	6.05 (0.66–55.32)	5.66 (0.61–52.14)	1.94 (0.19–18.99)	1.93 (0.19–19.04)
Allele						
T	Ref					
A	2.65⁣^∗^(1.63–4.27)	2.48⁣^∗^(1.52–4.04)				
rs17817449						
TT	Ref					
TG	2.32⁣^∗^(1.51–3.58)	2.12⁣^∗^(1.35–3.30)	1.73 (0.92–3.26)	1.65 (0.87–3.12)	2.84⁣^∗^(1.54–5.26)	2.85⁣^∗^(1.53–5.302)
GG	6.22⁣^∗^(2.43–15.96)	5.88⁣^∗^(2.24–15.40)	3.60⁣^∗^(1.11–11.63)	3.42⁣^∗^(1.04–11.14)	18.00⁣^∗^(2.27–242.2)	16.67⁣^∗^(2.1–13.39)
Dominant model						
TT	Ref					
TG + GG	2.65⁣^∗^(1.74–4.02)	2.47⁣^∗^(1.62–3.78)	1.95⁣^∗^(1.07–3.56)	1.85⁣^∗^(1.01–3.40)	3.38⁣^∗^(1.85–6.17)	3.36⁣^∗^(1.83–6.16)
Recessive model						
TT + TG	Ref					
GG	4.19⁣^∗^(1.67–10.50)	4.05⁣^∗^(1.59–10.29)	2.82 (0.90–8.80)	2.73 (0.87–8.59)	10.81⁣^∗^(1.39–83.68)	10.03⁣^∗^(1.29–77.99)
Allele						
T	Ref					
G	2.25⁣^∗^(1.63–3.12)	2.15⁣^∗^(1.54–2.99)				

*Note:* Model 1: unadjusted, Model 2: adjusted for gender and age.

Abbreviations: 95% CI, confidence interval; OR, odds ratio.

⁣^∗^*p* < 0.05.

**Table 6:** Differences in glucose and lipid parameters between FTO polymorphisms.

			Glu (mmol/L)	TC (mmol/L)	TG (mmol/L)	HDL (mmol/L)	LDL (mmol/L)
rs9939609	1	TT	5.14 (4.89∼5.44)	4.85 (4.39∼5.46)	0.99 (0.68∼1.41)	1.47 (1.27∼1.63)	2.9 (2.43∼3.47)
		TA + AA	5.33 (5.05∼5.57)	4.64 (3.99∼5.45)	1.68 (0.94∼2.57)	1.25 (1.05∼1.41)	2.79 (2.24∼3.40)
		*p*	0.003	0.080	<0.001	<0.001	0.130
	2	TT	5.11 ± 0.41	4.68 ± 0.78	0.80 (0.64, 1.23)	1.47 ± 0.27	2.77 ± 0.67
		TA + AA	5.35 ± 0.35	4.75 ± 0.93	1.84 (1.02, 2.90)	1.26 ± 0.25	2.78 ± 0.67
		*p*	0.002	0.654	<0.001	0.002	0.952
	3	TT	5.42 ± 0.82	5.19 ± 0.89	1.16 (0.83,156)	1.44 (1.23, 1.64)	3.16 ± 0.72
		TA + AA	5.37 ± 0.53	4.74 ± 0.93	1.54 (0.93, 2.45)	1.27 (1.03, 1.39)	2.87 ± 0.67
		*p*	0.679	0.003	0.002	<0.001	0.015

rs17817449	1	TT	5.12 (4.88∼5.48)	4.80 (4.40∼5.31)	0.80 (0.65∼1.13)	1.51 (1.35∼1.66)	2.85 (2.39∼3.30)
		TG + GG	5.21 (4.99∼5.49)	4.86 (4.13∼5.59)	1.44 (0.97∼2.09)	1.29 (1.11∼1.54)	2.94 (2.39∼3.53)
		*p*	0.102	0.632	<0.001	<0.001	0.349
	2	TT	5.13 ± 0.43	4.71 ± 0.72	0.80 ± 0.34	1.54 ± 0.26	2.76 ± 0.61
		TG + GG	5.19 ± 0.37	4.68 ± 0.90	1.80 ± 1.69	1.31 ± 0.25	2.77 ± 0.72
		*p*	0.200	0.797	<0.001	<0.001	0.779
	3	TT	5.40 ± 0.74	5.04 ± 0.91	1.04 ± 0.48	1.49 ± 0.28	3.06 ± 0.74
		TG + GG	5.41 ± 0.77	5.10 ± 0.93	1.83 ± 1.21	1.34 ± 0.29	3.11 ± 0.70
		*p*	0.929	0.670	<0.001	<0.001	0.610

*Note:* 1: total group, 2: ≤ 40 years group, 3: > 40 years group.

**Table 7:** Effect of haplotype on overweight/obesity and lipid parameters.

		OR	95% CI	*p*
GT	Overweight/obesity	1.81	(1.27–2.78)	<0.001
	TC	1.82	(1.24–2.69)	<0.001
	TG	4.43	(2.85–6.88)	<0.001
	HDL	2.32	(1.22–4.40)	0.010

GA	Overweight/obesity	2.99	(1.84–4.87)	<0.001
	TC	1.05	(0.66–1.68)	0.850
	TG	8.26	(5.08–13.49)	<0.001
	HDL	5.31	(2.90–9.73)	<0.001

*Note:p* values were adjusted for gender, age, weight, and BMI.

## Data Availability

The participants in the paper were from hospitals, and the data were not disclosed to protect patient privacy.

## Supporting Information

STROBE Statement—checklist of items that should be included in reports of observational studies.
